# Correlation of Splenic Parameters With Age in North Indian Population

**DOI:** 10.7759/cureus.41313

**Published:** 2023-07-03

**Authors:** Bhumica Dang, Akshi Walecha, Amit Garg, Yatin Talwar

**Affiliations:** 1 Anatomy, Bhagat Phool Singh Government Medical College, Khanpur Kalan, IND; 2 Cardiac Anesthesia, All India Institute of Medical Sciences, Jodhpur, IND; 3 Orthopaedics and Trauma, Maharaja Agarsen Hospital, Sonepat, IND; 4 Hospital Administration, All India Institute of Medical Sciences, Jodhpur, IND

**Keywords:** anthropometric measurements, correlation of spleen, forensic anthropometry, morphometry by spleen, age

## Abstract

Background: Estimation of age is of utmost importance in identifying the deceased. Research considering splenic morphometric parameters to determine the deceased's age is very rare in Haryana. The present study is done to find the correlation between the spleen's morphometric parameters and the deceased's age. The objective of the present study is to determine the changes in morphometry of the spleen with the age of the deceased.

Methods: The cross-sectional study was done in spleen specimens (56 males and 44 females) obtained from deceased adults aged 16-70 years belonging to North India from the Department of Forensic Medicine during autopsy from September 2021-June 2022. The data was analyzed using SPSS 20.0. Pearson's correlation test evaluated the association between splenic parameters and age. The correlation formula was derived to assess the significance of correlation.

Results: Correlation with age was negative and significant with splenic weight and splenic dimensions in both males and females. Mean splenic weight, length, breadth, and thickness were 113.83± 7.86 g, 10.2±1.04 cm, 6.53 ± 0.4 cm, 1.46 ± 0.074 cm respectively. Mean spleen weight, length, width, and thickness were 137 ±10.98g, 11.06 ± 1.28 cm, 6.9 ± 0.4cm, 1.66 ± 0.018 cm in males. Mean spleen weight, length, width, and thickness were 90.66 ± 4.74g, 9.34 ±0.8cm, 6.16 ±0.4cm, and 1.26 ±0.13cm in females.

Conclusions: Spleen weight and dimensions were higher in males than in females, and all the morphometric parameters increased up to the age of 45 and later decreased. The correlation of morphometric parameters of the spleen was negative and significant with age.

## Introduction

Age determination is an important forensic anthropological concern used in identifying unknown human remains. In events of mass disaster, setting off a criminal investigation, age determination is important as it can establish the correct identity of the deceased. Age determination is crucial evidence for investigators to conclude an individual's identity [1]. Age is considered the big four (besides sex, stature, and race) for the identification of the deceased. In 1888, Rollet was the first to study 50 female and 50 male corpses to show the relationship between body measurements and stature. Later Pearson derived the regression equations from the experimental results of Rollet, which he suggested were population specific. Many advancements have been since made to study the identification of the deceased [2]. 

The present study deals with the correlation of age with morphometric parameters of the spleen in the North-Indian population. The spleen is the largest unit of lymphoid tissue in the body. It is a soft, purple organ about the size of a fist in the left hypochondrium. It extends from the 9th-11th ribs with the long axis parallel to the 10th rib. The spleen is an intraperitoneal organ covered with peritoneum except at its hilum. The average dimensions are 12.5cm, 7.5 cm, and 2.5 cm in length, width, and thickness, respectively, and 150-200 g in weight [3]. The spleen develops as a lobulated mass from the mesoderm of the upper part of the dorsal mesogastrium under the cover of its left layer. The anterior part of the dorsal mesogastrium persists as a gastro-splenic ligament, and the posterior layer continues as a lie-no-renal ligament. Spleen has a unique architecture that plays a key role in the interactions between the circulatory, reticuloendothelial, and immune systems. [4]. Solomon Demissie in Southern Ethiopia studied morphometric assessment of spleen dimensions and correlates among individuals in Arba Minch town, Southern Ethiopia, in 708 individuals in February-March 2020. Their study was useful for forensic experts and anthropologists to find the deceased's age if the splenic specimen's parameters are known [3]. Sharma et al. studied the correlation of sonographic measurements of the spleen with age in the Nepalese population in 320 patients in the age group of 16-75 years in 2017 and found splenic length and thickness decreased with an increase in age in both males and females.

The study was conducted with the primary objective of studying the correlation between age and morphometric parameters of the spleen; the spleen was chosen because of its ease of measurement and handling [5].

## Materials and methods

The present study was carried out in the Department of Anatomy in collaboration with the Department of Forensic Medicine in North India from September 2021-June 2022. The cross-sectional study was done on 100 spleen samples (56 males and 44 females) aged 16-70 years belonging to North India. From the case records at the forensic department, an in-depth account of the deceased was gathered. The information gathered comprised age, sex, height, weight, and medical history. Ethical approval from IRB was taken prior to the beginning of the study. Approval was got under letter no 2022/1 dt 19 /5/2022.

Informed consent was taken from the relatives before the specimens were collected. The specimens were selected only from the fresh cadavers within twenty-four hours of death because after this duration, the morphometry is altered due to decomposition, and the spleen becomes pulpy, greenish steel, and gets reduced to a different mass [6]. Burn cases and deaths due to known diseases affecting the size, such as malaria, typhoid, miliary tuberculosis, HIV, Hepatitis, connective tissue disorders such as SLE, rheumatoid arthritis, thalassemia, polycythemia, lymphomas, and other malignancies have been excluded from the study. Exclusion criteria were decided from the history of the deceased.

Morphometric parameters

Length of the Spleen

**Figure 1:**
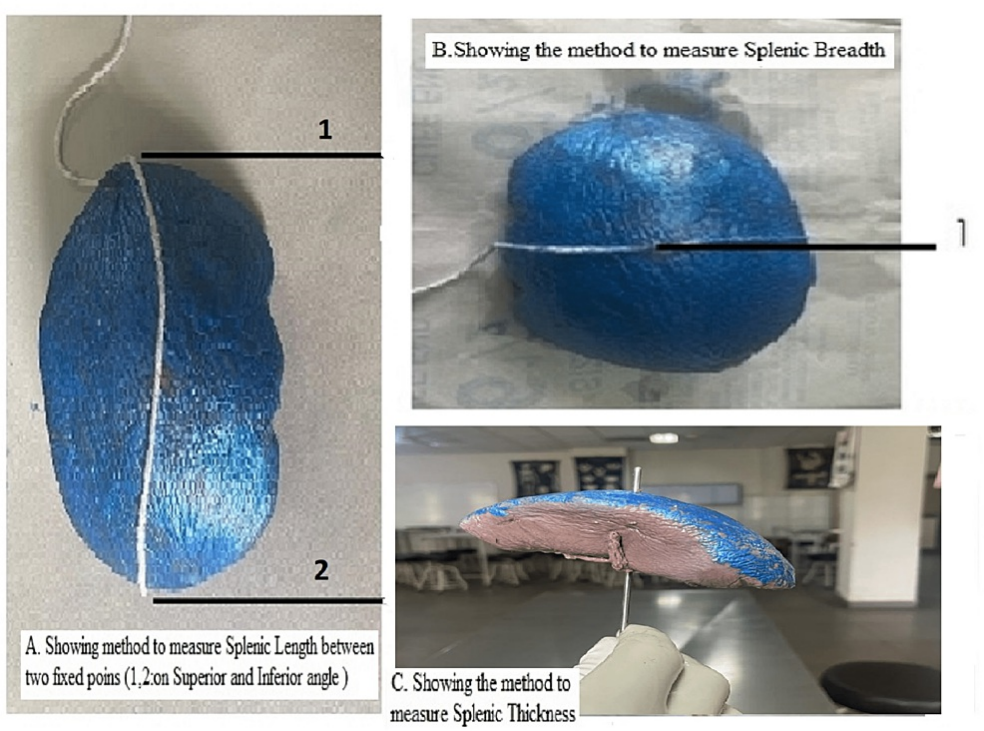
Method for calculating length (A) , Breadth (B) and Thickness (C) Length was calculated between two fixed points 1 and 2, Breath was calculated by taking mid point of splenic length as fixed point. Thickness was measured by inserting a needle at the maximum convexity and was then evaluated by measuring the length of the needle embedded inside

Length was determined on the diaphragmatic surface by a thread from the superior angle to the inferior angle passing through maximum convexity [7]. The measurement of length is shown in Figure 1.

Breadth of the Spleen

Breadth of the spleen was noted on the diaphragmatic surface by the thread passing horizontally through maximum convexity and the mid-point of the length [7]. The measurement of breadth is shown in Figure 1.

Thickness of the Spleen

Thickness was measured by inserting a needle at the maximum convexity and was then evaluated by measuring the length of the needle embedded inside [7]. The measurement of thickness is shown in Figure 1

Age of the Deceased

The Department of Forensic Medicine provided the documents pertaining to the deceased's age.

Statistical analysis

The splenic parameters and the deceased's age were noted carefully and recorded in a Microsoft Excel sheet, and data were analyzed using the Statistical Package for the Social Sciences (SPSS) software 20. Independent t-test was used to evaluate the differences between the variables. Pearson's correlation coefficient was used to assess the relationship between the spleen's morphometric parameters with the deceased's age. P< 0.05 was considered significant.

## Results

Demography

The research population was from North India, and spleens from 56 male and 44 female deceased individuals aged 16 to 70 years were gathered. The average age of the men was 37.16 ± 10.58 years (range: 17 -67 years), whereas the average female was 32.66 ± 7.00 years (range: 16 -69 years). The findings of participants' age and gender diversity in the study are depicted in Table 1.

**Table 1:** Participants' age and gender diversity in the study

Age (in years)	Male	Female	Total
16-25	10	5	15
26-35	12	14	26
36-45	13	11	24
46-55	12	10	22
Above 55	9	4	13
Total	56	44	100

Relationship between the deceased's age and splenic weight

The average weight of the spleen was 90.66 ± 4.74 g in women and 137± 10.98 g in men. Male spleens varied in weight from 120 g to 148 g, whereas female spleens varied in weight from 70 g to 115 g. Males and females displayed a moderately negative association with the splenic weight (r = -0.490) and a weakly positive connection (r = -0.264), respectively. The p-value for the correlation was 0.04 for men and 0.007 for women, as seen in Table 2.

**Table 2:** The relationship between the deceased's age and splenic weight Y is splenic weight in grams and x is age in years, p < 0.05 is considered statistically significant, p < 0.001 is considered statistically highly significant.

	Mean splenic weight in males(g)	Mean splenic weight in females (g)
Age 16-25	133 ± 10.4	91.3 ± 4.8
Age 26-35	138 ±10.9	92 ± 5.2
Age 36-45	145 ±12	111 ± 5.8
Age 46-55	142 ±11.4	88 ±4.1
Age >56	127 ±10.5	72 ±3.8
Splenic weight (g)	137 ± 10.98	90.66 ± 4.74
Correlation coefficient	-0.264	- 0.490
P value	0.04	0.0007
Regression equation	y = -0.322x + 142.29	y = - 0.4172 x +73.9

Correlation between the deceased's age with the length of the spleen

The mean splenic length in men was 11.06 ± 1.28 cm. The mean splenic length in females was 9.34± 0.8 cm. Male spleen sizes ranged from 10 to 13.4 cm, while female spleen sizes ranged from 8.5 to 10.5 cm. In men, splenic length and age of the dead had a negative connection (r= - 0.104). Splenic length in females had a negative connection with death age (r = -0.015). The p-value for the correlation was 0.002 for men and 0.03 for women, the p-value is significant for males and females. This implies that a correlation of splenic length with age is significant in both males and females. The same is shown in Table 3.

**Table 3:** Correlation between the deceased's age with the length of the spleen y is splenic length in cm and x is age of the deceased in years, p < 0.05 statistically significant; p <0.001 statistically highly significant.

	Mean splenic length in males (cm)	Mean splenic length in females (cm)
Age 16-25	10.5 ± 1.2	8.7 ±1.1
Age 26-35	11.2 ± 1.1	10.1 ± 0.9
Age 36-45	12.5 ± 1.8	10.2 ± 0.8
Age 46-55	10.8 ± 0.9	8.9 ± 1.2
Age >56	10.3 ± 1.4	8.8 ± 0.9
Splenic length (cm)	11.06 ± 1.28	9.34 ± 0.8
Correlation coefficient	- 0.104	- 0.014
P Value	0.002	0.03 (significant)
Regression equation	y= - 0.0145x + 10.96	y = - 0.0026 x + 9.83

Correlation of splenic breadth with age of the deceased

In males, the mean splenic breadth was 6.9 ± 0.4. In females, the mean splenic breadth was 6.16 ± 0.4 cm. Splenic breadth varied from 6- 8 cm in males, and in females, varied from 5.5 -7.2 cm. Correlation with age was negative in males (r = 0.368) and negative in females (r= - 0.014). The p-value for the correlation in males was 0.005, and in females was 0.03 (Table 4).

**Table 4:** Correlation of splenic breadth with age of the deceased

	Mean splenic breadth in males (cm)	Mean splenic breadth in females (cm)
Age 16-25	6.8 ± 0.7	5.8 ± 0.3
Age 26-35	7.4 ± 0.6	6.1 ± 0.2
Age 36-45	7.6 ± 0.2	6.7 ± 0.6
Age 46-55	6.6 ± 0.4	6.2 ± 0.5
Age >56	6.1 ± 0.1	6 ± 0.4
Splenic breadth (cm)	6.9 ± 0.4	6.16 ± 0.4
Correlation coefficient	- 0.368	-0.014
P value	0.005 (Significant)	0.03 (Significant)
Regression equation	Y= - 0.027x + 7.18	Y= -0.0019x + 5.25

Correlation with splenic thickness with age

The mean thickness of the spleen was 1.46 ± 0.074. In males, thickness varied from 1.66 ± 0.018 cm. In females, thickness varied from 1.26 ± 0.13 cm. Splenic thickness correlated with age negatively in males (r= - 0.169), and correlation was negative in females (r= - 0.089 p-values for correlation in males and females, respectively, were 0.02 and 0.002 (Table 5).

**Table 5:** Correlation of splenic thickness with age of the deceased

	Mean spleen thickness in males (cm)	Mean spleen thickness in females (cm)
Age 16-25	1.4 ± 0.01	1.2 ± 0.18
Age 26-35	1.8 ± 0.02	1.4 ± 0.12
Age 36-45	1.8 ± 0.01	1.3 ± 0.14
Age 46-55	1.7 ± 0.02	1.2 ± 0.12
Age >56	1.6 ± 0.03	1.2 ± 0.10
Splenic thickness	1.66 ±0.018	1.26 ± 0.13
Correlation coefficient	- 0.169	-0.089
P value	0.02	0.002
Regression equation	Y= - 0.0027x+ 1.7691	Y= - 0.0021x + 1.99

## Discussion

The present research was conducted to study the morphometric parameters of the spleen and its correlation with age in the North-Indian population. The correlation of splenic weight with age was negative in our study. Krumbhaar and Lippincott [8] studied variation in the weight of normal human spleen at different ages in 4000 cases from University Hospital and Philadelphia General Hospital. They found mean spleen weight was 155-160 gm between the ages of 26-65 years; after that, it fell markedly. Mccormick and Kashgarian [9] studied spleen samples in 284 Caucasians and 565 Negroes and found weight decreased with age in both races. Deland [10] studied the weight of the spleen in 440 samples at John Hopkins Hospital. They found spleen weight decreased between 20-29 years, followed by a relatively constant weight from 30-59 years and a decrease above 60 years. Myers and Segal [11] studied the weight of the spleen in 366 autopsies and found spleen weights decreased with age. Sprogoe -Jakobsen and Sprogoe -Jakobsen [12] studied spleen weights in 539 autopsies. Their study indicated spleen weight is not correlated with age apart from the fact that older people have smaller spleen weights. Ogiu et al. [13] studied splenic weight in 4,667 Japanese cases between 0-95 years and found average spleen weight reached a maximum at 19 years of age and decreased thereafter. Grandmaison et al. [14]studied the spleen weight in 684 adult autopsies in Caucasoid population for the year 1987-1991 and found spleen weight decreased with age. Kim et al. [15] studied healthy spleens in 526 Korean adults during post-mortem examination and found spleen weight reduced with increasing age.

In the present study, spleen weight correlated negatively with males' and females' age. The correlation coefficient was r = - 0.264 (p-value: 0.04) in males and r = - 0.490 in females (p-value: 0.011). The average length of the spleen in males was 11.06 ± 1.28 cm, and in females, it was 9.34 ± 0.8 cm. The correlation of length with age was negative in our study in males, r = - 0.104, and in females, r = - 0.014. The correlation was significant (p-value = 0.04) in males and females (p-value = 0.03).

Solomon et al. [3] studied the length of the spleen by ultrasonography in 708 individuals in Feb 2020 in Arba Minch town, Southern Ethiopia, and found that spleen length increased up to 40 years and decreased thereafter [3]. Arora et al. [16] examined 160 subjects and calculated splenic length decreases with an increase in age in both males and females. Asghar et al. [17] did a prospective study on 126 patients (72 males and 54 females) whose CT scan was normal and found that splenic length has a negative correlation with age. Chakarborti et al. [18] studied 2015 spleen length by USG in West Tripura in 146 healthy subjects (89 males and 57 females) and found spleen length decreased with increasing age in both males and females, and the correlation was significant. Singhal et al. [19] studied the measurement of the spleen about age in the adult Gujarati population in 2018 and found that length decreased with age in males and in females.

The average width of the spleen in males was 6.9 ± 0.4 cm, and in females, it was 6.16 ± 0.4 cm. The correlation of breadth with age was negative as r was -0.368 in males and r = -0.014 in females. The p-value was 0.005 in males and 0.03in females, so it was significant. Solomon et al. [3] his study found that the spleen breadth increased up to 40 years and thereafter decreased. Arora et al. [16] sonographically examined 160 subjects and calculated splenic width decreases with an increase in age in both males and females. In their CT study, Asghar et al. [17] found that width does not have a significant correlation with age. Singhal et al. [19], in their ultrasonographic study on the Gujarati population, found that in males and females, width increases up to 40 years and then decreases till 60 years.

The average thickness in the present study was 1.46 ± 0.074 cm in males and females. Correlation with thickness was negative in our study in both males and females. R-value was - 0.169 in males and - 0.089 in females. The p-value in males was 0.003, and in females was 0.002. Solomon et al. [3] found that spleen thickness increased up to 40 years and decreased thereafter. In a study by Arora et al. [16], both males and females decreased as age increased. In their CT study, Asghar et al. [17] found that splenic thickness does not have a significant correlation with age.

Singhal et al. [19], in their study on the spleen, found thickness decreased than increased, and in females, thickness increased and then decreased. Differences in spleen dimensions in the current study from studies conducted in other areas may be due to sample size differences, racial differences and nutritional status, and geographical differences [3]. As age advances, the spleen dimensions decrease due to a decrease in the number and size of B-cell follicles of the white pulp of the spleen, which occurs due to a decrease in the germinal center in older age groups.

In the present study, spleen dimensions increased to 45 years and decreased in old age. This is consistent with the study done in Western Nepal, Ethiopia, and Iraq (Table 6) [3].

**Table 6:** A comparative of all studies with summarization of all present and past studies. Dec: Decrease

Correlation of Age with		Krumbhaar and Lipincott [8]	Mccormick and Kashgarian [9]	Deland [10]	Myers and Segal [11]	Sprogoe -Jakobsen [12]	Ogiu et al [13]	Grandmaison et al [14]	Kim et al [15]	Asghar [17]	Arora et al [16]	Chakarborti S [18]	Singhal et al [19]	Solomon et al [3]	Present study
Year		(Year 1939)	(Year 1965)	(Year 1974)	(Year 1974)	(Year 1997)	(Year 1997)	(Year 2001)	(Year 2009)	(Year 2006- 2009)	(Year 2010)	(Year 2016)	(Year 2018)	(Year 2021)	2010-2014
Cases studied		4000	849	440	366	539	4667	684	526	126	160	146	500	708	100
Location		Philadelphia	Tennessee	Philadelphia		Denmark	Japan	France	South Korea	India (Delhi)	India (UP)	India (Tripura)	India (Gujrat)	Ethiopia	North-India
Weight	Male	Dec With Age	Dec With Age	Dec With Age	Dec With Age	Dec With Age	Dec With Age	Dec With Age	Dec With Age	N.A	N.A	N.A	N.A	N.A	Dec. with age
	Female	Dec With Age	Dec With Age	Dec With Age	Dec With Age	Dec With Age	Dec With Age	Dec With Age	Dec With Age	N.A	N.A	N.A	N.A	N.A	Dec. with age
Length	Male	N.A	N.A	N.A	N.A	N.A	N.A	N.A	N.A	N.A	N.A	Dec With Age	Dec With Age	Dec With Age	Increases up to 45 and then decreases
	Female	N.A	N.A	N.A	N.A	N.A	N.A	N.A	N.A	N.A	N.A	Dec With Age	Dec With Age	Dec With Age	Increases up to 45 and then decreases
Width	Male	N.A	N.A	N.A	N.A	N.A	N.A	N.A	N.A	N.A	N.A	N.A	Increased up to 40 years and after that decreased.	Increased up to 40 years and after that decreased.	Increases up to 45 and then decreases
	Female	N.A	N.A	N.A	N.A	N.A	N.A	N.A	N.A	N.A	N.A	N.A	Increased up to 40 years and after that decreased.	Increased up to 40 years and after that decreased.	Increases up to 45 and then decreases
Thickness	Male	N.A	N.A	N.A	N.A	N.A	N.A	N.A	N.A	Positive in males	Positive in males	N.A	Decreased then increased with age	Increases up to 40 and then decreases	Increases and decreases
	Female	N.A	N.A	N.A	N.A	N.A	N.A	N.A	N.A	Not significant in females	Not significant in females	N.A	Increases and then decreases.	Increases and then decreases	Increases and then decreases

Limitations

Regional Variations amongst the findings can be a limitation of the study; as the study was done in cadaveric samples of the northern region, variations may be seen when compared with findings of other regions. The researchers accept that digital measurements using CT and MRI could have provided more accurate data, and a larger sample size could have provided a more accurate correlation.

## Conclusions

Splenic dimensions and weight increased up to 45 years and then decreased. This correlation with age was significant in both males and females. The present study would prove important in identifying the deceased's age in mass murders and other forensic investigations. 

In regions not equipped with the latest and/or advanced techniques, such as molecular techniques, this method can be used as a conventional method, especially in mass disasters/ homicides, for identification.
